# Are Chest Radiographs Routinely Indicated After Chest Tubes Placed for Non-Surgical Reasons Are Removed?

**DOI:** 10.7759/cureus.7339

**Published:** 2020-03-20

**Authors:** Raiko Diaz, Krunal B Patel, Patricia Almeida, Saketh P Shekar, Felix Hernandez, Jinesh PpP Mehta

**Affiliations:** 1 Pulmonary Medicine, Aventura Hospital and Medical Center, Aventura, USA; 2 Pulmonary and Critical Care, Cleveland Clinic Florida, Weston, USA; 3 Pulmonary and Critical Care, Aventura Hospital and Medical Center, Aventura, USA

**Keywords:** pneumothorax, chest tube, intensive care unit

## Abstract

Background

The insertion and subsequent removal of chest tubes are frequently performed procedures for the management of pneumothoraces, pleural effusions, and cardio-thoracic surgical interventions. A chest radiograph is commonly obtained after the removal of a chest tube to rule out the interval development of a pneumothorax. This practice has been questioned in various retrospective and prospective studies conducted on surgical patient populations, showing little to no benefits in performing routine chest X-rays (CXRs) after chest tube removal unless clinical symptoms such as worsening respiratory status and hemodynamic compromise are present.

Material and Methods

A four-year retrospective study was conducted using the Cleveland Clinic Foundation database. A chart review was performed, and 1,032 patients were screened, with 200 patients meeting inclusion criteria. The inclusion criteria included patients who underwent chest tube insertion for non-surgical reasons. The primary outcome was the percentage of clinically significant pneumothoraces detected by routine CXR after chest tube removal.

Results

Out of the 200 patients included in the study, 53 had a CXR after chest tube removal showing a residual pneumothorax. Out of the 53 patients, 50 ended up not needing chest tube re-insertion, as the patients were asymptomatic and hemodynamically stable. Only three patients required chest tube re-insertion due to respiratory symptoms and significant hemodynamic changes after the chest tubes were removed. In all three cases, the symptoms manifested prior to the CXRs being obtained; therefore, the decision to reinsert each chest tubes was made based on clinical signs rather than imaging. As expected, the practice of repeating CXRs after removal of the chest tubes resulted in delayed discharges despite patients reporting no symptoms and being hemodynamically stable.

Conclusions

Our study findings correlate with prior smaller studies on surgical patients. Symptoms and hemodynamic data seem to be a better predictor of whether a patient will require chest tube re-insertion or not. Routine CXR after chest tube removal also leads to prolonged hospital stay.

## Introduction

The history of tube thoracotomy (the insertion of a tube in the chest) dates back to ancient Greece, with Hippocrates describing its use for the treatment of empyema [1]. Since then, tube thoracotomy has been widely used in medicine, with the influenza epidemic of 1917 (empyema) and the Korean war (post-trauma care) being two notable examples [1].

There are various indications for the use of tube thoracotomy, such as spontaneous pneumothorax, trauma-related chest injuries, infection leading to empyema, pleural effusions of various etiologies, and post-surgical management. Evidence-based guidelines exist to guide physicians on when and how to insert a chest tube [2]. There are also data supporting chest radiography to confirm the placement of the chest tube as well as management thereafter. However, there are not enough data or specific guidelines when it comes to the removal of the chest tube and the need for chest radiography post-removal to assess for possible pneumothorax [3].

It is a conventional medical practice at most institutions to obtain a chest radiograph a few hours after the chest tube is removed. This practice has been questioned in various retrospective and prospective studies, showing little to no benefits in performing routine chest radiographs after the removal of the chest tube. Moreover, routine post-removal chest radiographs lead to a significant increase in resources and radiation, as well as unnecessary interventions based on imaging [4].

In 2002, Pacharn et al. published one of the first retrospective studies related to this subject [5]. The study was performed in a pediatric post-cardiac surgery population and concluded that clinical signs and symptoms identified nearly all patients with clinically significant pneumothoraces. Furthermore, Palesty et al. published a five-year retrospective study including surgical and trauma patients and concluded that chest radiography following the removal of chest tubes should not be a routinely performed procedure [6]. More recently, in 2010, Goodman et al. published a retrospective study including trauma patients [7]. Results once again showed that selective omission of chest radiographs following chest tube removal in less severely injured, asymptomatic, non-ventilated patients does not adversely affect outcomes or increase re-intervention rates.

Although most of the data come from studies performed on surgical and trauma patients, it stands to reason that non-surgical cases would likely yield similar results. The purpose of our study was to use a retrospective chart review to determine the percentage of clinically significant pneumothoraces detected by routine chest radiograph after chest tube removal in non-surgical patients admitted to the hospital and whether incidental findings of clinically insignificant pneumothoraces on imaging affected the length of stay. We hypothesized that the practice of performing routine chest X-ray after the removal of chest tubes in non-surgical patients does not result in an improved rate of detection of clinically significant pneumothoraces and may lead to an increased length of stay.

## Materials and methods

This retrospective medical chart review was performed using data from the Cleveland Clinic Foundation. Institutional Review Board (IRB) approval was obtained from the Cleveland Clinic's IRB prior to starting data collection, and data were de-identified using numeric codes to assure patient confidentiality.

The chart review was performed for all admissions during the years 2012-2016 that required chest tube insertion. In total, 1,032 patients required insertion of a chest tube for any reason while in the hospital. Inclusion criteria for our dataset were patients over the age of 18 and those requiring chest tube placement for non-surgical/trauma indications. Patients who underwent surgical intervention prior to chest tube removal were excluded from the study. Those who did not have a chest radiograph after the chest tube was removed were also excluded. Patients who were discharged from the hospital with the chest tube or expired prior to the chest tube being removed were also excluded from the study. The primary outcome of the study was to determine the percentage of clinically significant pneumothoraces detected by routine chest radiograph after chest tube removal. The secondary outcome was to determine whether routine chest radiograph delayed discharge in asymptomatic patients with residual pneumothoraces not requiring intervention after chest tube removal. A total of 200 patients met both the inclusion and exclusion criteria.

All patients included had a chest radiograph obtained within 36 hours of chest tube removal. The size of the pneumothorax was recorded as documented by the radiologist reading the report. Unfortunately, we did not have the imaging available for every patient and therefore could not make that determination ourselves. 

Data collected included the following: age, sex, BMI, admitting diagnosis, medical history, smoking status, duration of chest tube prior to removal, vital signs, complications of removal, size of pneumothorax (if present) on post-chest tube removal radiograph, whether patients were symptomatic, requirement of chest tube re-insertion, and chest tube placement indication.

Data were collected from the time of chest tube placement to discharge or expiration date of the patient. Chest tube re-placement was only documented if it had to be performed within 72 hours of removal.

Statistical analysis included calculation of frequencies and means with standard deviations of the variables, one-way analysis of variance for each variable to determine the effect, Pearson’s correlation between values, and p-values for testing differences between subpopulations.

Access to the data was only available to the investigators at Cleveland Clinic Florida. All of the data were collected and analyzed at Cleveland Clinic Florida. Microsoft Excel was used to collect and analyze the data on a password-protected file stored on a secure network drive. The abstract for this study was presented as a poster presentation at the American Thoracic Society 2019 International Conference.

## Results

**Figure 1:**
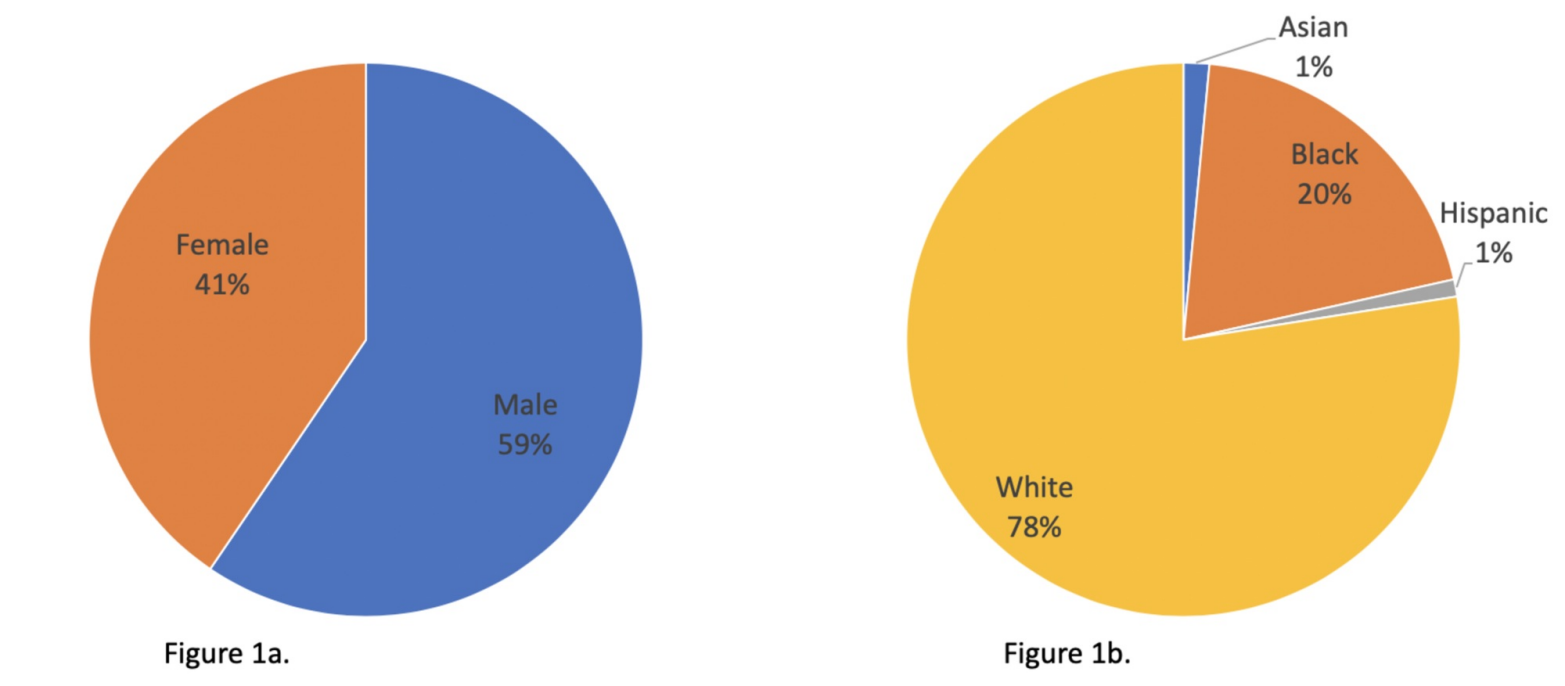
Patient’s demographics. (a) Gender. (b) Ethnicity.

A total of 200 patients met the inclusion criteria in our retrospective chart review. Of these, 59% were males and 41% were females (Figure 1). The mean age was 60 years, whereas the mean BMI was 24.9 (Table 1). A majority of the patients were Caucasian (78%), with African-Americans accounting for 20% of the patients (Figure 1). The length of time that the chest tubes stayed in place averaged 5.1 days, ranging from 1 day to 43 days.

**Table 1:** Patient's demographics

	N	Minimum	Maximum	Median	Mean	Standard Deviation
Age	200	18	96	62.0	59.9	19.8
BMI	200	14.0	44.4	24.1	24.9	5.33

All patients had a chest radiograph performed within 24 hours of the chest tube removal. Nursing notes, medical staff notes, and vital signs were then reviewed to identify those patients who experienced complications, symptoms, or vital sign changes after the chest tube was removed.

**Figure 2:**
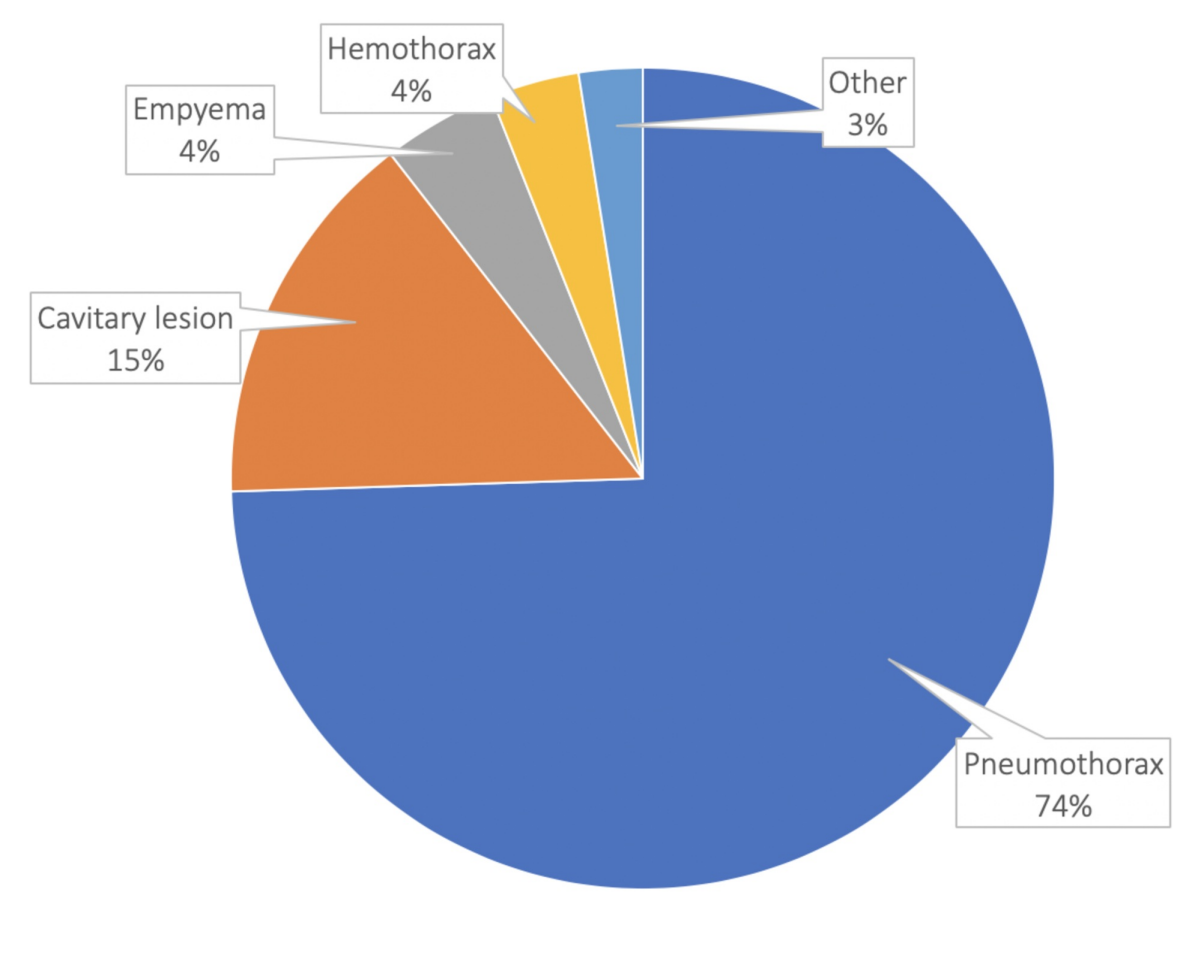
Indications for chest tube placement

The most common indication for chest tube placement in our study was pneumothorax (74.5%) followed by cavitary lesions (15%), empyema (4.5%), hemothorax (3.5%), and pleural effusions (2.5%) (Figure 2). Most patients had a history of smoking (67%), hypertension (52.5%), or chronic obstructive pulmonary disease (50.5%) (Table 2).

**Table 2:** Medical co-morbidities COPD, chronic obstructive pulmonary disease

Co-Morbidity	Percentage of Patients
History of smoking	67
Hypertension	52.5
COPD	50.5
Hyperlipidemia	27
Coronary artery disease	22
Diabetes mellitus	20
Anemia	14.5
Congestive heart failure	14
Liver disease	8
Chronic kidney disease	7
History of stroke	7

A total of 53 patients of the 200 included in our study had a pneumothorax on chest radiograph after the chest tube was removed. Out of the 53 patients who had a residual pneumothorax, only 3 were symptomatic or had changes in vital signs. Two of the patients were awake and able to communicate, whereas one of them was on mechanical ventilation and unable to communicate. The two patients who were able to communicate expressed symptoms of worsening dyspnea and chest pain within two to six hours after the chest tube was removed. The patient who was on mechanical ventilation was unable to communicate; however, hemodynamic changes were noted within three hours of chest tube removal. For all three patients, the most common changes in hemodynamics were an increase in heart rate as well as respiratory rate. Two of the patients were found to have a large pneumothorax, whereas the third patient had a moderate-sized pneumothorax. The symptoms/signs preceded imaging in all three cases. These patients were the only three patients who required intervention after chest tube removal. In all three cases, this involved re-insertion of the chest tube. The decision to re-insert the chest tube was made based on clinical symptoms/signs, and imaging was used as an adjunct to confirm the decision (Table 3). The Pearson correlation value between symptoms, hemodynamic changes, and the need for intervention was R = +1, which indicates a very strong correlation.

**Table 3:** Demographics and indications for chest tube re-insertion CP, chest pain; HD, hemodynamic; COPD, chronic obstructive pulmonary disease

Race	Age	BMI	Reason	Duration	History	Symptom	Size	Smoker
White	31	22.4	Spontaneous pneumothorax	2	Alpha-1 antitrypsin	Dyspnea, CP, tachycardia within two hours	Large	Yes
White	36	24.1	Traumatic pneumothorax	8	None	HD changes Within three hours	Moderate	Yes
Black	60	28.1	Spontaneous pneumothorax	3	COPD	Dyspnea around six hours after removal	Large	Yes

The patients who had a residual pneumothorax on radiography after chest tube removal but had no symptoms or changes in hemodynamics did not require intervention. This group of patients was managed conservatively, in most cases with oxygen supplementation. These patients, who had a residual pneumothorax but no symptoms, stayed in the hospital for longer than those who did not have a pneumothorax on chest radiograph after chest tube removal. On average, they stayed for 0.98 days ± 0.78 days after chest tube removal as compared to the group that did not have a pneumothorax (0.15 ± 0.38 days ) on radiograph after chest tube removal (p<0.00001).

## Discussion

Our data support the observations of prior studies on surgical patients. Routine chest radiographs after chest tube removal in asymptomatic patients fail to demonstrate any benefit and prolong hospital stay. At our institute, every patient who developed clinically significant pneumothorax requiring intervention also had symptoms or hemodynamic changes. Based on our results, symptoms or changes in vital signs are a better predictor of whether a patient will require intervention than routine chest radiograph.

Cost-effective medicine and the emergence of managed care have significantly changed the way we practice medicine. Nowadays, all medical participants are more aware of cost containment in the health care field. Chest X-ray cost varies from institution to institution. Across the country, the average for an inpatient chest X-ray and radiologist interpretation averages close to $400. When this charge is multiplied by the number of X-ray films after chest tube removals, the cost becomes substantial. We must also keep in mind that those patients who had a clinically insignificant pneumothorax likely underwent a repeat X-ray to monitor for resolution. Such patients usually end up staying in the hospital for longer, waiting for resolution or to establish stability of the pneumothorax. In our study, those patients who had a clinically insignificant pneumothorax on X-ray ended up staying for 0.98 days compared with 0.15 days after chest tube removal. Such prolongation of hospital stay is sure to add to the cost of health care of our patients and tax payers.

Another consideration is the amount of radiation that patients as well as health care personal are exposed to. Although the amount of radiation exposure has become comparatively small, it still carries some risks [8]. It could be argued that one life-threatening complication identified by routine chest radiograph justifies other unnecessary chest X-rays performed. However, such life-threatening complications are rare and usually present with significant symptoms as well as hemodynamic changes that are detected and acted upon more rapidly by clinical evaluation than by waiting for a chest radiograph.

Our study certainly has limitations. One of the limitations is the fact that this is a retrospective study. Due to the nature of the study itself, we were unable to standardize certain aspects of the study, such as the technique used to remove the chest tube, as well as radiology read and size of the pneumothorax. Another weakness of the study is the small sample size. It was difficult to screen patients who underwent chest tube placement for non-surgical reasons as well as those who did not undergo certain intervention such as video-assisted thoracoscopic surgery or pleurodesis prior to the chest tube being removed. It must also be emphasized that the vast majority of our study population comprised white males with a history of smoking; therefore, we were not able to generalize these results to a more diverse population.

Only three patients required intervention, re-insertion of the chest tube, after chest tube removal. These findings support the fact that the incidence of pneumothoraces after chest tube removal is very rare. It is also important to emphasize that a total of 53 patients in this study had a pneumothorax on radiography after removal of the chest tube; however, only three of them had symptoms and required intervention. This supports our hypothesis that the vast majority of patients having a chest tube removed do not need a repeat X-ray after removal.

## Conclusions

The results of our study correlate with similar studies on patients who required chest tube placement for surgical reasons. Routine chest radiographs are not necessary for all patients who have a chest tube removed. Only those patients who have clinical evidence of complications after removal of the chest tube should undergo a chest radiograph. Critical patients on mechanical ventilation or those who are unable to communicate likely will also continue to require chest radiograph after chest tube removal as clinical evaluation is limited in those patients. Our study also suggests that performing routine chest X-ray on every patient increases the length of hospital stay and also the number of chest radiographs performed, and therefore the amount of radiation exposure. Our study, like every other study performed on this subject, was limited by the sample size and the small number of pneumothoraces requiring intervention. However, to our best knowledge, this is the first study evaluating the need for routine chest X-ray after chest tube removal in a medical, non-surgical population. More studies with a larger sample population, or perhaps a prospective randomized clinical trial, are warranted to further evaluate this topic.
